# Phytochemical Profiles, In Vitro Antioxidants and Antihypertensive Properties of Wild Blueberries (*Vaccinium angustifolium*)

**DOI:** 10.3390/foods14244281

**Published:** 2025-12-12

**Authors:** Deborah O. Omachi, Thaniyath Shahnaz, Brandon Gines, Norma Dawkins, John O. Onuh

**Affiliations:** 1 Department of Food and Nutritional Sciences, Tuskegee University, 1200 W. Montgomery Rd, Tuskegee, AL 36088, USA; domachi3100@tuskegee.edu (D.O.O.); tshahnaz1460@tuskegee.edu (T.S.); ndawkins@tuskegee.edu (N.D.); 2 Department of Chemistry, Tuskegee University, 1200 W. Montgomery Rd, Tuskegee, AL 36088, USA; bgines@tuskegee.edu

**Keywords:** free radicals, antioxidants, antihypertensive, polyphenolic, phytochemical

## Abstract

Cells generate free radicals as part of their normal physiological process. However, the production of excessive amounts of free radicals is a key contributor to various pathological conditions as an imbalance between oxidants (reactive oxygen/nitrogen species, ROS) and antioxidants leads to oxidative stress. Blueberries have bioactive properties that could reduce oxidative stress, blood pressure, and lipids in people at risk of chronic diseases associated with metabolic syndrome. The aim of this study, therefore, is to identify the bioactive phytochemicals in blueberries and determine the in vitro antioxidant and anti-hypertensive properties. Total phenolic content, total flavonoid content, radical scavenging, and angiotensin-1 converting enzyme inhibitory activity of freeze-dried blueberry powder were determined using standard methods. The results showed that the identified polyphenolic compounds include quercetin, gallic acid, cyanidin chloride, vitamin C, trans caffeic acid, procyanidin B1, and procyanidin B2. Blueberry samples exhibited significantly higher ACE-inhibitory activity (*p* < 0.05) of 32.7 ± 0.6%, 34.5 ± 4.7%, and 56.2 ± 2.0% at different concentrations of 0.1 mg/mL, 0.5 mg/mL and 2.0 mg/mL and also an increasing radical scavenging activities of 66.4 ± 2.9%, 72.3 ± 2.0%, and 83.4 ± 0.5% with increased concentrations at 1.0 mg/mL, 2.0 mg/mL, and 4.0 mg/mL, respectively. It had a high total phenolic and flavonoid content of 741.11 ± 5.0 mg gallic acid equivalents [GAE]/g) and 679.2 ± 5.0 mg catechin equivalents/g), respectively, at a concentration of 15 mg/mL. The results showed that blueberries are rich sources of bioactive compounds with significant antioxidants and antihypertensive properties that could benefit health, suggesting that they could be an important source of functional ingredients for potential nutraceutical development.

## 1. Introduction

Scientific research has demonstrated that berries serve as an excellent source of phytochemicals, which have been found to possess restorative and promotive properties for human health [1]. The most consumed berries are strawberries, blueberries, blackberries, raspberries, cranberries, red currants, black currants, chokeberries, wolfberries, huckleberries, and lingonberries [2,3]. The incorporation of berries into one’s diet has emerged as a crucial aspect of nutritional enhancement [4,5]. The blueberry (*Vaccinium* sp.) belongs to the family Ericaceae (commonly known as the heath family), which also encompasses the cranberry, azalea, and rhododendron. These berries are characteristically small, blue-black, and fruit-shaped, originating from Northern Europe and North America [6]. This plant is indigenous to the United States and Canada, occurring spontaneously in hilly and forested areas. The availability of different types of blueberries is dependent on the growing season and harvesting time. These include highbush blueberry plants (*Vaccinium corymbosum* L.), the rabbit-eye blueberry (*Vaccinium ashei Reade*), lowbush blueberries, the blueberry plants or wild blueberry (*Vaccinium angustifolium Aiton*), and bilberry (*Vaccinium myrtillus* L.) [7,8]. There is no doubt that blueberries possess the greatest antioxidant capacity and are therefore regarded as an essential functional food ingredient [9]. The fruits are typically transformed into a variety of products, including jam, syrup, pie, soup, tart, cobbler, smoothie, pancake, muffin, cupcake, salsa, salad, lemonade, waffles, ready-to-eat breakfast cereals, yogurts, and beverages [10]. Blueberries are of particular interest due to their high antioxidant content, which is the result of their diverse chemical composition, including flavonoids and phenolic acids with potential functional properties [11]. The berries are renowned as an abundant source of nutrition, encompassing fiber, tannins, anthocyanins, proanthocyanidins, vitamin C, ellagic acid, omega-3 fatty acids, carotenoids, and minerals [12,13]. The bilberry (*Vaccinium myrtillus*) is a European wild blueberry that contains a higher concentration of anthocyanins (ACNs) than cultivated blueberry species [14,15], which contributes to its reputation as a nutritious fruit. Blueberries are a rich source of carbohydrates, vitamins, and minerals [16,17]. Blueberries also represent a valuable source of dietary fiber, with a concentration of 3–3.5% of fruit weight [8]. Moreover, blueberries exhibit an elevated content of several phytochemicals, including ascorbic acid and phenolics. A considerable number of the proposed advantageous health effects linked to blueberry consumption can be attributed to the bioactive properties of the phytochemical constituents. The potential benefits of blueberries for human health have received significant attention recently, largely due to these bioactive components [18]. Nevertheless, despite these advances, significant gaps remain in our understanding of the potential of anthocyanins and other polyphenols in CVD prevention [19,20]. Furthermore, the existing literature lacks information regarding the biomarkers of hypertension and oxidative stress, as well as the mechanisms underlying the in vitro antioxidant and antihypertensive effects of blueberries. There is also limited data on the oxidative stress-reducing property of blueberries. Therefore, the objective of this research is to determine the phytochemical profiles, in vitro antioxidants, and antihypertensive properties of Wild Blueberries (*Vaccinium angustifolium*).

## 2. Materials and Methods

### 2.1. Materials

The freeze-dried blueberry powder used for the study was obtained from Z Natural Foods, FL, USA. All the solvents, reagents, and standards used were of analytical grade. The standards (gallic acid, cyanidin chloride, procyanidin B1, procyanidin B2, quercetin, ascorbic acid, delphinidin chloride, trans caffeic acid, and resveratrol) were purchased from Sigma Aldrich, Inc. (St. Louis, MO, USA). ELISA kits were purchased from R&D Systems, Inc. (Minneapolis, MN, USA). All solvents and water used for HPLC-UV measurements were of HPLC grade. 2, 2 diphenyl-1 picrylhydrazyl radical (DPPH), Folin–Ciocalteu phenol reagent, gallic acid, catechin, glutathione, captopril, angiotensin converting enzyme, ACE (EC 3.4.15.1) from rabbit lung were purchased from Sigma Aldrich (Sigma Chemicals, St. Louis, MO, USA). N-[3-(2-Furyl) acryloyl]-Phe-Gly-Gly, 98% (FAPGG) was purchased from MedChem Express (Monmouth Junction, NJ, USA).

#### 2.1.1. Standard Preparation

The phenolic reference compounds were accurately weighed and dissolved in either water or alcoholic solvents (typically ethanol) depending on Pubchem solubility information. All were diluted to a concentration of 2.5 mg/mL and mixed with internal standard leucine enkephalin (LeuEnk) to create a 250 µg/mL solution of reference compound with 960 µg/mL LeuEnk.

##### Sample Extraction

The blueberry sample extraction was conducted in accordance with the methodology outlined by [21], with slight modifications. A total of 2 g of the sample was weighed and transferred into a glass test tube. Subsequently, 10 mL of the extraction solvent (HPLC grade 80% methanol) was added with the aid of a graduated cylinder, with precision in measuring the 10 mL volume maintained. The sample was subjected to brief high-speed vortexing for 10 s and subsequent sonication for 30 min at 40 °C and 40 kHz. Subsequently, the sample was subjected to centrifugation at 2000× *g* for a period of five minutes. Following this, the suspension was filtered using Whatman filter paper and a glass funnel, and the resulting solution was transferred to a glass vial. The supernatant was then decolorized with HPLC-grade water and filtered through a 13 mm syringe filter with a 0.2 µm PTFE membrane, before being transferred to an HPLC vial. Sample solutions with a concentration of 200 mg/mL were mixed with an internal standard to create a final concentration of 960 µg/mL LeuEnk in the solution. A single concentration of standard was spiked into the sample to verify the identity and retention time of the standard compounds in the samples.

##### Preparation of Blueberry Extract

One gram of freeze-dried blueberry powder was dissolved in 10 milliliters of 70% ethanol solution. The extract was filtered using Whatman No. 1 filter paper. The filtrate was subsequently collected and subjected to drying by means of a rotatory evaporator (Buchi Rotavapor R-205 equipped with a self-cleaning dry vacuum system, TM model 2025, Buchi, New Castle, DE, USA), operating at 40 °C for a period of 15 min. Thereafter, the extract was stored at −20 °C [22].

##### Characterization of Blueberry Extracts by LC/MS Analysis

The analysis was performed on a Vanquish UHPLC system (Thermo Fisher, Waltham, MA, USA), which was coupled with a quadrupole orbitrap mass spectrometer (Orbitrap Exploris 240, Thermo Fisher, Waltham, MA, USA) with electrospray ionization (H-ESI) according to a method described by [23] with slight modifications. The analyses were conducted in both positive and negative modes, and the data was processed with the Xcalibur software (V4.4.16.14). Injection of 1 μL of the sample was made on a C18 column (Accucure RP-MS, 2.1 µm, 2.1  ×  100 mm, Thermo Fisher, Waltham, MA, USA) with a 200 μL/min flow rate of mobile phase solution A (99.9% water with 0.1% formic acid) and solution B (95% acetonitrile 5% water with 0.1% formic acid). The gradient began at 0% B, held for 3 min, followed by a linear ramp to 100% B at 9 min, held for 1 min, then back to 0%B for a total analysis time of 15 min. The flow was diverted to waste for the first minute of analysis. The mass spectra, MS was set to small molecule with scan range at 70–700 *m*/*z* with resolution of 120,000, standard AGC target, 70% RF lens, maximum injection time auto, with EASY-IC on. The spray voltage was 3500 V in positive and 2300 V in negative mode, the ion transfer tube temperature was 280 °C, and the vaporizer temperature was 250 °C. All the phenolic compounds were identified by comparing their retention times and exact mass with those of the standard compounds (vitamin C, quercetin, gallic acid, cyanidin chloride, delphinidin chloride, trans caffeic acid, procyanidin B1, procyanidin B2). Standard curves and sample concentrations were calculated with Xcalibur software. The amount of each phenolic compound was expressed as µg/g of dry mass [24,25,26].

##### DPPH (2,2-Diphenyl-1-picrylhydrazyl) Radical Scavenging Assay Blueberry Polyphenolic Extracts

The scavenging activity of blueberry extracts against DPPH radicals was determined using a previously described method [27], which was modified for use in a 96-well clear flat-bottom plate. The DPPH radical was diluted in a 50 mL volumetric flask with methanol, and the resulting solution was used to prepare the DPPH stock. The appropriate diluted sample at different concentrations (1 mg/mL, 2 mg/mL, and 4 mg/mL) and the DPPH stock solution were then added to a 96-well clear flat-bottom plate in the ratio of 40 µL and 200 µL, respectively. Absorbance values for the blanks and samples were determined at 517 nm following a 30 min incubation period using a microplate reader (SpectroStar Nano; BMG Labtech, Ortenberg, Germany). A lower absorbance of the reaction mixture is indicative of a higher free radical scavenging activity. The free radical scavenging activity was measured as the amount of extract required to decrease the initial absorbance (517 nm) of the DPPH radical concentration by 50% (IC50) in comparison to the control, according to the following equation. The percentage of DPPH radical scavenging activity of the extracts was determined using the following equation.

$$\mathrm{DPPH}\;(\%)=\frac{{Ab}_{s(blank)}-{Ab}_{s(samples)}}{{Ab}_{s(blank)}}\times 100$$

##### Determination of Total Phenolic Content (TPC)

The total phenolic content (TPC) of the blueberries was measured using a modified Folin–Ciocalteu colorimetric assay, as previously described by [28]. Briefly, 12.5 µL of approximately diluted sample was added to 50 µL of distilled water. Then, 12.5 µL of Folin–Ciocalteu phenol reagent was added to the mixture. After 5 min, 125 µL of 7% Na_2_CO_3_ solution was added to the mixture. Prior to spectrometric analysis, the samples were incubated for 90 min in the dark at 25 °C. The absorbance of the diluted solution was measured at 750 nm compared to a blank consisting of all the reaction agents except the extract using a microplate reader (SpectroStar Nano, BMG LABTECH, Ortenberg, Germany). A standard curve for total phenolics was developed using a gallic acid standard solution. The results were expressed as milligrams of gallic acid per 100 g. The results are expressed as mean (mg gallic acid equivalents g^−1^ dry blueberries) ± SEM for 3 replications.

##### Determination of Total Flavonoid Content (TFC)

The total flavonoid content (TFC) was determined with a colorimetric method [29]. Briefly, 0.25 mL of 80% methanolic extract was diluted with 1.25 mL of distilled water. Then 75 µL of a 5% NaNO_2_ solution was added, and the mixture was allowed to stay at room temperature. After 6 min, 150 µL of a 10% AlCl_3_ × 6H_2_O solution was added, and the mixture was allowed to stand for another 5 min. After that, 0.5 mL of 1 M NaOH was added. The solution was well mixed, and the absorbance was measured immediately against a blank at 510 nm using a microplate reader (SpectroStar Nano, BMG LABTECH, Ortenberg, Germany) in comparison with the standards prepared similarly with known (±) catechin concentration. Then the results were expressed as mg of catechin equivalents.

##### ACE-Inhibitory Activity of Blueberry Polyphenolic Extracts

The ability of the polyphenolic extracts to inhibit the in vitro activity of ACE was measured according to a spectrophotometric method using N-[3-(2-Furyl) acryloyl]-Phe-Gly-Gly, 98% (FAPGG), as the substrate, which is employed as a chromogenic probe for quantitative detection of ACE activity [30]. Briefly, 1 mL of 0.5 mM FAPGG (dissolved in 50 mM Tris–HCl buffer containing 0.3 mM NaCl, pH 7.5) was mixed with 20 µL of ACE (1 U/mL; final activity of 26 mU) and 200 µL of 1 mg/mL sample (dissolved in the same buffer) in a 96-well microplate. The decreased absorbance (∆Abs) at 345 nm, due to cleavage of the Phe- Gly peptide bond of FAPGG, was recorded for 30 min at 37 °C using a microplate reader. The buffer was used instead of a sample solution in the blank (uninhibited) reaction mixture.

ACE inhibition was calculated as follows:

$$ACE\;Inhibition\;(\%)\frac{Slope(Blank)-Slope(sample)}{Slope(blank)}\times 100$$

##### Statistical Analyses

A minimum of triplicate assays was used to find out the mean values and standard deviations. For statistical analysis, analysis of variance has been used, while significant differences (*p* < 0.05) between mean values were determined by Duncan’s multiple range tests. The IBM SPSS statistical package (version 22) was used for all statistical analyses.

## 3. Results and Discussion

### 3.1. Phytochemical Profile

The use of UHPLC-LC/MS for chemical analysis is regarded as an effective method, as it allows for the selective detection of compounds, high levels of sensitivity, and the potential to achieve accurate and precise identification [31]. Therefore, it has recently been employed extensively for the identification of bioactive compounds present in natural products [32]. The current study investigates the presence of a range of phytochemicals in blueberries, including anthocyanins, flavonoids, and phenolic acids. The dominant polyphenolic compounds identified in the blueberry extracts are presented in Table 1. The identified compounds were vitamin C, quercetin, gallic acid, cyanidin, trans caffeic acid, procyanidin B1, and procyanidin B2. The presence of gallic acid (2.172 ± 0.02 μg/mL), procyanidin B1 (1.915 ± 0.02 μg/mL), and vitamin C (1.655 ± 0.02 μg/mL) in concentrations exceeding (*p* < 0.05) those of the other compounds may be a significant contributing factor to the bioactive and antioxidants properties of this extract [33,34,35]. Delphinidin was below the detectable limit, hence it was not found. These findings align with those of previous studies that have documented the presence of these constituents in the same sample grown under different climatic conditions in diverse geographical regions [36,37,38,39]. The anthocyanins present in our blueberry extract were comparable to those previously reported in other blueberry species and their jam extracts [40,41,42,43]. However, the results obtained by [35] identified four cyanidin and one petunidin glycosides as anthocyanin-based pigments and five quercetin glycosides with neochlorogenic acid and chlorogenic acid as hydroxycinnamic acids in aronia. This disparity may be attributed to differences in berry type, processing technique, or the standards used.

In other studies, 12 distinct anthocyanins were identified in both blueberry and its jam extract [13,44]. These included three derivatives of delphinidin, three of cyanidin, two of petunidin, one of peonidin, and three of malvidin, all with the 3-O-glucoside, 3-O-galactoside, or 3-O-arabinoside substitutions. The divergence in the observed trends may potentially be attributed to inherent differences in the species, cultivation, and extraction techniques employed for the various blueberry samples under consideration.

### 3.2. DPPH Radical Scavenging Activities

**Figure 1:**
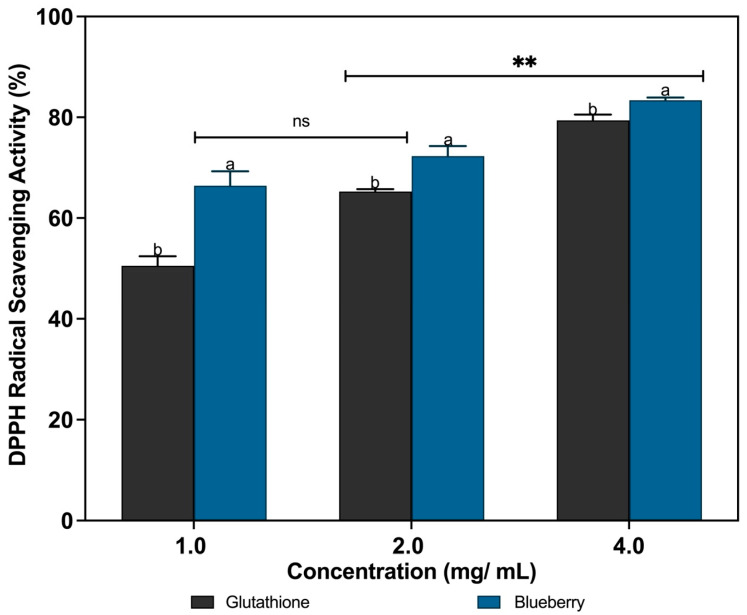
In vitro DPPH radical scavenging activities of blueberry and glutathione. Bars are mean ± standard deviation. ^a,b^ Bars that do not share a letter (superscript) are significantly different (*p* < 0.05) within the same concentration. ns: no significant difference between different concentrations; ** significant difference between different concentrations. Spectrophotometer wavelength at 517 nm.

DPPH has been documented to be a stable synthetic compound utilized for the assessment of free radical scavenging activity across a range of antioxidants [45]. Upon interaction with free radicals generated during oxidative reactions, antioxidants facilitate the formation of stable byproducts that effectively halt the oxidation process [46]. In the presence of a DPPH molecule, an antioxidant capable of donating a hydrogen atom will neutralize the stable free radical, resulting in a change in the absorption spectrum. This process can be spectrophotometrically quantified with a maximum absorbance at 517 nm in methanol, and is widely employed for testing reducing substances, particularly natural compounds [46]. Consequently, this approach offers an invaluable means of evaluating antioxidant potential. Figure 1 depicts the results of the analysis of the DPPH radical scavenging activity of the blueberry extracts at various concentrations. A significant increase in the percent scavenging activity was observed with increased concentrations (*p* < 0.05), from 66.4 ± 2.9%, 72.3 ± 1.9% and 83.4 ± 0.5% at 1.0 mg/mL, 2.0 mg/mL, and 4.0 mg/mL, respectively. This is attributed to the scavenging effects of blueberry extracts, which resulted in a higher number of hydrogen atoms and electrons that could be donated. The antioxidants were found to be capable of reducing the radical DPPH to the yellow-colored diphenyl-picrylhydrazyl. This method is based on the reduction in a methanolic DPPH solution in the presence of hydrogen-donating antioxidants, which results in the formation of a non-radical form, DPPH-H [27].

The DPPH radical scavenging ability of the extracts was significantly higher (*p* < 0.05) than that of glutathione, which was used as a standard antioxidant compound in this study. The extracts demonstrated a DPPH radical scavenging activity (DRSA) of over 50% at all concentrations tested, with the blueberry extracts exhibiting the highest activity (Figure 1). Similar results were obtained by [47], who previously observed increased DPPH scavenging activity in basil leaves due to the scavenging effect of basil extracts. Different accessions of basil exhibited maximum antioxidant activity at varying concentrations. Additionally, our findings are consistent with the conclusions of a previous study conducted by [48], which indicated that all fruit extracts (including blueberry, jackfruit, blackberry, black raspberry, red raspberry, strawberry, and California table grape) demonstrated an increase in radical scavenging activity with increased concentrations, where the highest percentage of DPPH radical inhibition was observed in samples containing blueberry, black, and red raspberry extracts, with concentrations ranging from 20 to 100 µg/mL. The inhibition rates for these samples ranged from 38.5% to 87.9%, 64.2% to 89%, and 37.6% to 87%, respectively. However, a study by [49] indicates that the DRSA was not wholly concentration-dependent in the case of various vegetable extracts. This is evidenced by the observed decline in the value at the highest concentration (1 mg/mL) for most of the extracts. A decreased scavenging capability at 1 mg/mL may be attributed to pro-oxidative effects or polyphenol aggregation. This phenomenon may be attributed to the occurrence of polyphenol aggregation through hydrophobic interactions at high concentrations, resulting in a reduction in the number of binding sites and interactions with the DPPH free radical. These findings agree with a previously published report that similarly demonstrated the presence of decreased DRSA when polyphenol concentrations exceeded 0.5 mg/mL [50].

### 3.3. The Total Phenolic Content (TPC)

**Figure 2:**
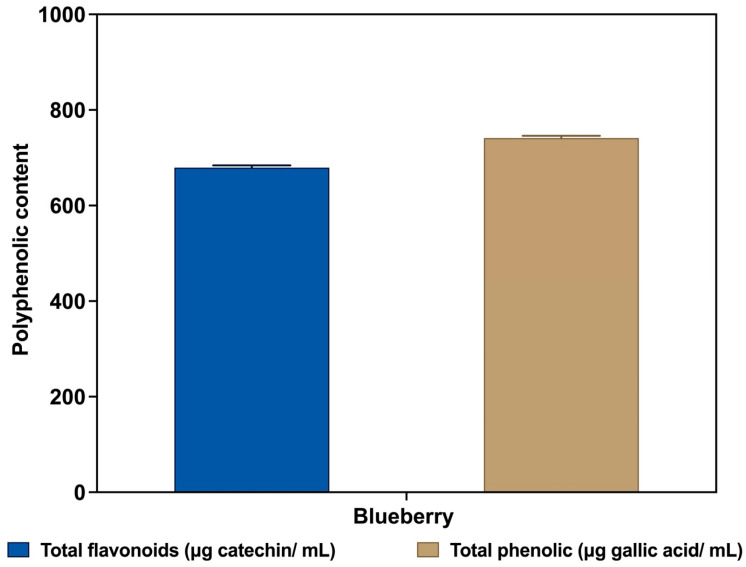
Total flavonoid and total phenolic content of blueberry (15 mg/mL). Spectrophotometer wavelength at 510 nm and 750 nm, respectively. Bars are mean ± standard deviation.

TPC is a marker of blueberry antioxidant capacity and is typically utilized as an indicator of antioxidant potential. Among polyphenolic compounds, flavonoids are regarded as the most predominant, with catechin, genistein, quercetin, epicatechin, luteolin (or epigenin), butein, and naringenin serving as illustrative examples [51]. Therefore, flavonoid content is a significant parameter for estimating polyphenol accumulation by plants and represents the most crucial quality index for antioxidant activity. Hence, it is advantageous to determine phenolics and flavonoids in blueberry extracts. Our investigation demonstrates a high total phenolic content of 741.11 ± 5.0 mg gallic acid equivalents (GAE) per gram at 15 mg/mL (Figure 2). A similar outcome was observed by [52], who reported that the total phenolic content of *Vaccinium corymbosum* blueberries is 709.92 mg gallic acid equivalent. Additionally, a study by [48] reported that the total phenolic content of fruit extracts exhibited a range of 250.1 ± 17.12 to 965.6 ± 2.9 mg GAE/g. The total phenolic content of the fruit extracts under consideration in this study was found to be as follows: the highest concentration was found in black raspberries, followed by blueberries, jackfruit, red raspberries, California table grapes, blackberries, and finally, strawberry. The TPC data obtained from previous findings revealed values between 251 and 310 mg GAE/100 g for cultivated blueberries and 577–614 mg GAE/100 g for wild Italian blueberries [53]. In a recent report by [40] a TPC of 519.95 ± 4.56 (mg/100 g FW) was determined in *Vaccinium uliginosum* L. Similarly, the TPC values reported for blueberry extracts analyzed falls within a range of 424.84–819.12 mg GAE/100 g fresh weight [54]. Also, other researchers investigated the TPC of wild Vaccinium species [55]. The researchers reported that the TPC of these species varied considerably, ranging from 489 to 702 mg per 100 g. The difference observed in TPC is attributable to disparate geographical locations and the fact that the biosynthesis of phenolic compounds is influenced by a range of abiotic and biotic factors, including temperature fluctuations, irradiation, herbivory, and pathogenic infection [56]. Also, disparate quantification methodologies (encompassing the utilization of distinct standards), as well as additional elements such as the genus, species, and cultivar of the plants employed, in addition to environmental disparities at the point of plant cultivation, such as climatic conditions, soil composition, light exposure, infestation by pests, and the maturation stage of the fruit, in addition to the manner of handling and storage of the fruit [57]. Additionally, phenolics display quantitative variations at different genetic levels within species [54].

### 3.4. The Total Flavonoid Content (TFC)

The present study demonstrates a considerable high TFC of 679.2 ± 5.0 mg catechin equivalents per gram at a concentration of 15 mg/mL (Figure 2) when compared to previous studies that have reported total flavonoid content in various fruit extracts, with [48] citing values of 151.7 ± 1.1 mg QE per gram for blueberry extracts and 186.4 ± 1.7 mg QE per gram for blackberry extracts. Ref. [52] demonstrated a catechin equivalent concentration of 165.48 mg per 100 g of fresh weight. The results of [58] indicate that the highest (*p* < 0.05) TFC was observed in Kangkong (305.39 ± 2.93), while stem amaranth (62.22 ± 0.24) exhibited a significantly lower value. In a study conducted by [54], the total flavonoid content was found to be 110.36 ±12.3 mg QE/100 g and 112.50 ±15 mg QE/100 g in different species of *Vaccinium myrtillus*. The discrepancies in flavonoid content across studies may be attributed to the use of diverse varieties and varying standards (catechin versus quercetin) for quantifying total flavonoids in fruit extracts, the type of extraction solvent used because some of the previous studies used solvents and not water as well as the reporting methodology (Fresh Weight versus Dry Weight) employed. The results collectively indicate that habitual consumption of these vegetables and fruits may be a significant contributor to flavonoid intake. This finding is noteworthy because prior research has demonstrated a direct relationship between flavonoid intake and reduced risks of chronic diseases, including stroke, cancers, and other forms of cardiovascular disease [59,60,61,62].

### 3.5. In Vitro ACE-Inhibitory Activity

**Figure 3:**
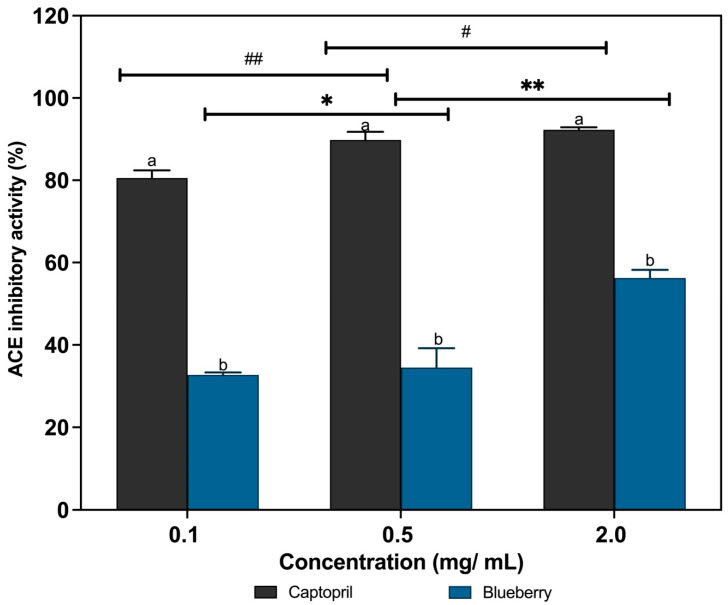
In vitro ACE-inhibitory activities of blueberry and Captopril at 345 nm. Bars are Mean ± standard deviation. Bars with different letters are significantly different at *p* < 0.05. ^a,b^ Bars that do not share a letter (superscript) are significantly different (*p* < 0.05) within the same concentration. * No significant difference between different blueberry concentrations; ** Significant difference between different blueberry concentrations; ^#^ No significant difference between different captopril concentrations; ^##^ Significant difference between different captopril concentrations.

The degree of ACE inhibition exhibited by the blueberry extracts varied according to their respective concentrations. Figure 3 illustrates that blueberries exhibited a significantly higher ACE-inhibitory activity (*p* < 0.05) at different concentrations, 32.7 ± 0.5%, 34.5 ± 4.7%, and 56.2 ± 2.0% at concentrations of 0.1 mg/mL, 0.5 mg/mL, and 2.0 mg/mL, respectively. However, the blueberry extracts demonstrated a significantly (*p* < 0.05) weaker inhibition when compared to captopril (80.5 ± 1.9%, 89.8 ± 2.0%, 92.3 ± 0.6%), the ACE-inhibitory drug. This may be attributed to captopril’s meticulously designed molecular structure, which enables a considerably stronger and more targeted interaction with the active site of ACE, as opposed to plant compounds, which often possess less selective binding and comparatively weaker inhibitory effects due to their intricate chemical composition and potential for multiple active sites [63]. The findings of the current study are comparable to those of a prior investigation conducted by [64], wherein the ACE inhibition capacity of *Syzygium polyanthum* Wight (Walp.) leaves was evaluated. It exhibited the highest inhibition activity of 69.43 ± 0.60% and the standard drug captopril demonstrated a 74.95% ACE inhibition at 2.06 ng/mL. A study conducted by [65] demonstrated that pepsin-pancreatin-hydrolyzed pea protein (PPHPp) exhibited superior angiotensin-converting enzyme inhibition, with a percentage of 61.82%. PPHPp demonstrated a pronounced antihypertensive impact, exhibiting an immediate systolic blood pressure decrease of −26.12 mmHg within 2 h post-oral administration. Furthermore, the findings align with those of [57], where different levels of ACE inhibition were attained for the vegetable extracts, namely ash gourd (76.51% ± 0.25%), brinjal (72.48% ± 0.02%), and snake gourd (66.82% ± 0.99%). A significant difference in inhibitory strength (*p* < 0.05) was observed between the vegetable extracts and captopril. Furthermore, this could be due to its high potency and specificity; captopril exhibits a high potential for ACE inhibition. This allows it to effectively block the enzyme at relatively low concentrations, whereas plant-based ACE inhibitors often require higher doses to achieve comparable outcomes, which can be attributed to their lower level of specificity [66,67,68]. Additionally, the blueberry extracts exhibited diminished ACE inhibition relative to captopril, a finding that aligns with the robust antihypertensive efficacy associated with conventional pharmaceutical agents. The reduced risk of negative side effects associated with natural products may make them preferable antihypertensive agents in comparison to synthetic drugs [63]. Thus, incorporating these plant extracts into food products for regular consumption may be an effective method for preventing hypertension [57].

The substantial antioxidant characteristics of these fruits, which are potentially ascribed to their phenolic compounds, and the ACE inhibition assay, which appraised their anti-hypertensive capacity, imply that blueberry extracts may function as valuable constituents for the formulation of functional foods, as demonstrated in this study. In comparison to other literature, this study has been expanded to in vivo research and subsequently a human clinical trial.

### 3.6. Conclusions

The phytochemical profile of freeze-dried blueberry powder assessed through UHPLC-LC/MS analysis indicated the presence of anthocyanins, flavonoids, and phenolic acids. The identified polyphenolic compounds included quercetin, gallic acid, cyanidin chloride, vitamin C, delphinidin chloride, trans-caffeic acid, procyanidin B1, and procyanidin B2. Mass spectrometry analysis confirmed the identification of the bioactive phytochemical compounds, as indicated by their distinctive *m*/*z* ratio. This comprehensive data therefore implies that blueberry extracts comprise a rich source of bioactive compounds and could exhibit considerable potential as a natural source of metabolites with biological activities for the development of functional ingredients in nutraceuticals and supplements that are beneficial to human health. The present study has demonstrated that blueberry extracts possess the ability to scavenge ROS. The antioxidant properties of these fruits are likely attributable to their phenolic compounds. An ACE inhibition assay is a common test used to evaluate the antihypertensive potential of pharmaceuticals and plant extracts. In this study, the ACE inhibition activity was found to be highly active, suggesting that blueberry extracts may serve as valuable components for the development of functional foods and nutraceuticals aimed at mitigating oxidative stress-induced diseases.

### 3.7. Future Perspectives

The considerable range of phytochemicals present in blueberries, coupled with their capacity for therapeutic application, renders them a promising category of functional food. Further investigation into the mechanisms of action of these substances, their therapeutic applications, and the necessity for standardized clinical studies to establish appropriate dose guidelines and guarantee long-term safety will elucidate their role in promoting human health and preventing illness. It would be beneficial to conduct additional studies that consider more standards to identify bioactive compounds and phytochemical profiles, as well as more reliable biomarkers.

## Figures and Tables

**Table 1:** Concentration of individual bioactive compounds in blueberries.

Polyphenolic/Bioactive Compounds	Molecular Formula	Actual Retention Time (min)	*m*/*z* (Da)	MS/MS (Da)	Concentration (μg/mL)
Vitamin C	C_6_H_8_O_6_	1.1	175.0239– 175.0257	70.0000– 700.0000	1.655
Cyanidin	C_15_H_11_O_6_^+^	8.8	287.0536– 287.0564	70.0000– 700.0000	0.795
Quercetin	C_15_H_10_O_7_	9.2	303.0484– 303.0514	70.0000– 700.0000	0.283
Gallic Acid	C_7_H_6_O_5_	2.4	169.0135– 169.0151	70.0000– 700.0000	2.172
Procyanidin B2	C_30_H_26_O_12_	8.7	577.1323– 577.1381	70.0000– 700.0000	0.565
Procyanidin B1	C_30_H_26_O_12_	8.5	577.1323– 577.1381	70.0000– 700.0000	1.915
Trans Caffeic Acid	C_9_H_8_O_4_	8.9	179.0340– 179.0358	70.0000– 700.0000	0.368

## Data Availability

The entirety of the data collected during the course of this study is incorporated within the scope of this published article. Should additional information become necessary, it may be obtained from the corresponding author upon submission of a request that is deemed reasonable.

## Abbreviations

GAE	Gallic acid equivalents
ROS	Reactive oxygen species
RNS	Reactive nitrogen species
ELISA	Enzyme-linked immunosorbent assay
HPLC	High-performance liquid chromatography
ACE	Angiotensin-II converting enzyme
ACNs	Anthocyanins
H-ESI	Electrospray ionization
UPLC	Ultra-high performance liquid chromatography
DPPH	2,2-diphenyl-1-picrylhydrazyl
DRSA	DPPH radical scavenging activity
TFC	total flavonoids content
FAPGG	N-[3-(2-furyl) acryloyl]-Phe_Gly_Gly, 98%
LCMS	Liquid chromatography/mass spectrometry
